# microRNA-199a-5p regulates epithelial-to-mesenchymal transition in diabetic cataract by targeting SP1 gene

**DOI:** 10.1186/s10020-020-00250-7

**Published:** 2020-12-04

**Authors:** Xin Liu, Qiaoyun Gong, Longfei Yang, Min Liu, Lingzhi Niu, Lufei Wang

**Affiliations:** 1 Eye Center, The Second Hospital of Jilin University, #218 Ziqiang Street, Changchun, Jilin China; 2 Department of Ophthalmology, Shanghai General Hospital (Shanghai First People’s Hospital), Shanghai Jiaotong University Medical School, #100 Haining Road, Shanghai, China; 3 Jilin Provincial Key Laboratory On Molecular and Chemical Genetics, The Second Hospital of Jilin University, #218 Ziqiang Street, Changchun, Jilin China

**Keywords:** Diabetic cataract, MiR-199a-5p, Epithelial-to-mesenchymal transition, Specific protein 1

## Abstract

**Background:**

As a common ocular complication of diabetes mellitus, diabetic cataract is becoming a leading cause of visual impairment. The progression of diabetic cataract progression involves epithelial-to-mesenchymal transition (EMT), the precise role of which remains to be investigated. As microRNAs (miRNAs) are suggested to be involved in the pathogenesis of many diseases, identification of aberrantly expressed miRNAs in diabetic lens epithelial cells (LECs) and their targets may provide insights into our understanding of diabetic cataract and potential therapeutic targets.

**Methods:**

Diabetic cataract capsules and LECs exposed to high glucose (25 mmol/L, 1–5 days) were used to mimic the model. Quantitative RT-PCR was performed to evaluate the differential expression of miRNA. Dual luciferase reporter assay was used to identify the binding target of miR-199a-5p. The expression of EMT-associated proteins was determined by immunofluorescence and Western blot analysis.

**Results:**

Our results showed the differential expression of miR-9, -16, -22, -199a and -204. MiR-199a was downregulated in diabetic cataract capsule and hyperglycemia-conditioned human LECs. Specific protein 1 could be directly targeted and regulated by miR-199a in LECs and inhibit EMT in diabetic LECs.

**Conclusion:**

Our findings implied miR-199a could be a therapeutic target by regulating SP1 directly to affect EMT in diabetic cataract and provided novel insights into the pathogenesis of diabetic cataract.

## Background

Diabetes mellitus (DM) is a kind of endocrine diseases that seriously endanger human health and the incidence is increasing globally. As the common ocular complication of DM, diabetic cataract, as well as diabetic retinopathy, becomes the leading cause of visual impairment for the incidence and progression is increasing in patients with DM (Pollreisz and Schmidt-Erfurth 2010). Diabetic cataract often manifests earlier and severer than the age-related cataract (ARC), in which hyperglycemia seems to play a promoting role, including activation of the aldose reductase (AR) receptor, accumulation of sorbitol and galactositol and advanced glycation end-products(AGE), and oxidative stress damage actived by reactive oxygen species (ROS) in LECs (Oishi et al. (2006); Varma et al. 2012; Martinez and Iongh 2010; Peppa and Vlassara 2005). The pathogenesis of diabetic cataract is a multifactorial process and altered expression of genes related to development, proliferation, differentiation, apoptosis, autophagy and epithelial-mesenchymal transition (EMT) in LECs could contribute to cataract (Wang et al. (2017); Worgul et al. 1989). EMT is the trans-differentiation of epithelial cells into mesenchymal cells. During the process, LECs lose their normal conditions of morphology and transcriptional program, then, attain phenotype characteristics of mesenchymal cells, including enhanced migratory capacity, invasiveness, resistance to apoptosis and increased production of extracellular matrix component (Iongh et al. 2005; Han et al. 2018). The occurence of EMT in epithelial cell has been reported in the formation of diabetic cataract in vivo and in vitro Zhang et al. (2017a). Aldose reductase and AGE is found to be associated with proliferation and EMT of lens epithelial cells (Zablocki et al. 2011; Wu et al. 2020).

However, the detailed molecular mechanisms of EMT regulation in diabetic cataract still need to be explored. It is suggested that noncoding RNAs are also playing critical roles in diabetic cataract development, in which microRNAs (miRNAs) are of great interest in mechanistic studies. miRNA is a large group of endogenous, small non-coding RNAs of 20–25 nucleotides in length. miRNA regulates target gene expression post-transcriptionally by binding to the 3′-untranslated region of the target (3′-UTR), inducing mRNA degradation or translational repression. miRNAs play regulatory roles in many cellular processes, such as cell differentiation, proliferation and apoptosis, and now are found to participate in the pathogenesis of many ocular diseases (Yu et al. 2017; Peng et al. 2012; Wang et al. 2014). Studies on cataract have focused on miRNAs expression changes to identify potential functional miRNAs and their target genes (Wei and Sun 2019; Zou et al. 2018). Acoording to these studies, some distinctly altered miRNAs including miR-9, -15a, -16, -22, -23, -29, -124, -135b, -138, -145, -195, -199a, and -204 would be further investigated in this study. Based on the network of miRNA-mRNA-protein working in the progression of diabetic cataract, a taget of miRNA would also be identified to affect EMT through the study.

Specific protein 1 (SP1), a member of the SP/Kruppel-like factor super-family (Sp/KLF family) of transcription factors, is required for the transcription of many housekeeping genes (Black et al. 2001). SP1 is related to multiple cell behaviors, such as proliferation, migration and EMT. It is reported to associate with aggressive behavior, invasive clinical phenotype and poor clinical outcomes in various cancers. In the lens epithelial cells, SP1 was reported to work as an essential transcription factor, participating in oxidative stress injury and regulating α-crystallin expression (Liu et al. 2016; Chhunchha et al. 2018). Sp1 binding site in the Slug promoter was reported to be responsible for TGF-β-induced Slug expression and EMT in patients with cataracts (Choi et al. 2007). Meanwhile, TGF-β could promote EMT of LECs under HG conditions (Han et al. 2018).

In the present study, we intended to analyze the alterations of miRNAs in hyperglycemic LECs or diabetic cataract capsules. Furthermore, we investigated the regulatory effects of miR-199a on SP1 in EMT of LECs, hoping to help in mechanism researches and the development of therapeutic strategies for diabetic cataract.

## Methods

### Clinical samples collection

Fresh anterior capsules of lens samples were obtained from 33 patients (18 male, 15 female; aged from 52 to 60 years) with ARC, and 33 patients (16 male, 17 female; aged 45–56 years) with diabetic cataract who underwent phacoemulsification at Eye Center of Second Hospital of Jilin University between December 2016 and November 2018.This study followed the tenets of the amended Declaration of Helsinki and was approved by the Ethic Committee of the hospital and conducted with the approval of the institutional review board (IRB) at Jilin University. All samples were collected with informed consent from the patients. All patients underwent a complete preoperative ophthalmologic examination and lens capsule samples were obtained by intact continuous curvilinear capsulorhexis, without vascular contact or damage to the iris or other intraocular structures. Patients without DM were placed in one group as control, and patients with DM but without proliferate diabetic retinopathy were placed in the other group as diabetic cataract. All patients were with type 2 DM, and all patients had grade III cortical cataracts according to the Lens Opacities Classification System III (Chylack et al. 1993). Patients who had ocular surgery or ocular disease other than DR were excluded from the study.

### Cell culture, hyperglycemia treatment and transfection

Human lens epithelial cell line SRA01/04 was obtained from American Type Culture Collection (ATCC, Manassas, VA, USA). Cells were incubated in low glucose DMEM (Hyclone, Beijing, China) containing 10% FBS (Gibco; Thermo Fisher Scientific, Waltham, MA, USA) at 37 °C in a humidified atmosphere containing 5% CO_2_.

SRA01/04 cells were plated at 3000 cells/cm^2^ in 6-well plates (Corning, USA) and treated with normal glucose (NG; 5.5 mmol/L) as a control (Additional file 1: Fig. S.A) or with high glucose (HG; 25 mmol/L) for 1, 3, and 5 days (Additional file 2: Fig. S.B).

The hsa-miR-199a-5p mimic, mimic control, miR-199a-5p inhibitor and inhibitor control were chemically synthesized by GenePharma (Shanghai, China). The sequence of miR-199a-5p mimic was 5′-cccaguguucagacuaccuguuc-3′ and the sequence of miR-199a-5p inhibitor was 5′-gggucacaagucugauggacaag-3′. Cells, with 70–80% confluence in 6-well plates, were transfected with miRNA mimic/inhibitor or control by Lipofectamine RNAiMAX (Invitrogen, USA) according to the manufacturer’s instructions. After transfections for 48 h or 72 h, the cells were harvested for mRNA and protein analysis, respectively.

Human SP1 specific siRNA and negative siRNA were chemically synthesized by Sangon Biotech (Shanghai, China). The cells were transfected using lipofectamine™ 2000 (Invitrogen) for 6 h before the medium was changed. For further analysis, the cells were cultured for 48 h. The sequences of SP1 siRNA were as follows: 5′-CUAUGAACUACAGGUGUUU-3′, and a non-silencing siRNA with sequence 5′-AGUCUCCACGUGUACGUTT-3′ was used as the negative control.

### Total RNA Isolation and Quantitative RT-PCR

To extract total RNA from capsules collected from patients and LECs under hyperglycemia, an Eastep Super total RNA extraction kit (Promega, China) was used according to manufacturer protocol. RNA concentration and purity were measured by NanoDrop 2000c spectrophotometer (Thermo Fisher Scientific). RNA samples with an A260/A280 value of 1.8–2.0 were used for further analysis and the integrity of RNA samples was assessed by 1% agarose-gel electrophoresis.

Total RNA of miRNA was polyadenylated and reverse transcribed with an All-in-One miRNA first-strand cDNA synthesis kit (GeneCopoeia, USA). To analyze miRNA-expression levels, All-in-One miRNA quantitative PCR detection kit with specific primers (GeneCopoeia) was applied on a LightCycler 480 (Roche Diagnostics), and the Hsn-U6 was used as internal control. For mRNA analysis, the total RNA was reverse transcribed using a Perfect real-time RT reagent kit (Takara Bio, China), and then qPCR was performed on a LightCycler 480 (Roche Diagnostics). The relative expression levels of miRNAs and mRNAs were calculated using the 2^−ΔΔCt^ method, in which the ratio of expression between an experimental group and the control group was determined.

### Western blot

Total proteins were collected using a cell-lysis buffer (Thermo Fisher Scientific, USA) following the manufacturer’s instructions and concentrations were determined with bicinchoninic acid protein assay kit (Beyotime, China). Then the proteins were assayed with SDS–polyacrylamide gels and transferred to polyvinyl difluoride membranes (Thermo Fisher Scientific). The membranes were blocked and incubated with primary antibodies against β-actin (1:1000; CMC-TAG, USA), SP1 (1:500; Santa Cruz Biotechnology, USA), alpha-SMA (1:500; Cell Signaling Technology (CST), USA), E-cadherin (1:200; CST, USA), FSP-1 (1:500; ABclonal, China) and vimentin (1:1000; CST, USA). Quantification of Western blots was processed using Image Pro Plus software (Media Cybernetics Inc., USA).

### Immunofluorescence

The anterior capsule with epithelial cells was fixed immediately in 0.1 mol/L phosphate buffer containing 4% paraformaldehyde and stored at 4 °C. The fixed anterior capsules were rinsed and blocked with 1% BSA. The capsules were incubated at room temperature for 4 h with alpha-SMA (1:500; CST) for 4 h, E-cadherin (1:100; CST) for 2 h, FSP-1 (1:50; ABclonal) and vimentin (1:50; CST) for 4 h and incubated with their appropriate secondary antibody conjugated with Alexa Fluor 633 or 488 (1:800; Thermo) at 37 °C for 1 h. After wash, Hoechst staining (Sigma, diluted to 1:5000) was performed to visualize the nuclei. After mounting, samples on the glass slides were photographed by a confocal microscope (Olympus FV1000, Japan) for further analysis. The objective used was 60× (Oil. PlanApo N, Olympus), NA = 1.42. Zoom = 1. These images were single optical images rather than maximum intensity projections. The fluorescence intensity was calculated by Image J software.

### Luciferase reporter assay

Human SP1-3′UTR including conserved binding sites for miR-199a-5p, as well as the mutation, was amplified by PCR, and inserted into the XhoI/NotI-digested vector pSI-Check2 (Hanbio Biotechnology, China). The cells were transiently transfected with the pSI-Check2 vectors including 3′-UTR regions or mutant 3′-UTR regions of SP1, miRNA mimic, and NC mimic. After transfection for 48 h, luciferase activity was determined using a Dual-Glo™ Luciferase Assay System (Promega). Firefly luciferase activity was normalized to that of Renilla luciferase for each sample.

### Statistical analysis

The Statistical Package for Social Sciences software (SPSS Inc., USA) was applied for statistical analyses. Each experiment was repeated at least three times, and all the data are presented as means ± standard deviation. Analysis of differential expression was performed using an unpaired *t*-test or Mann–Whitney U tests, while multiple groups were analyzed by one-way ANOVA or Kruskal–Wallis tests. A *p* value below 0.05 was considered to be statistically significant.

## Results

### EMT and miRNA expression changes in human diabetic cataract and hyperglycemic LECs

**Fig. 1:**
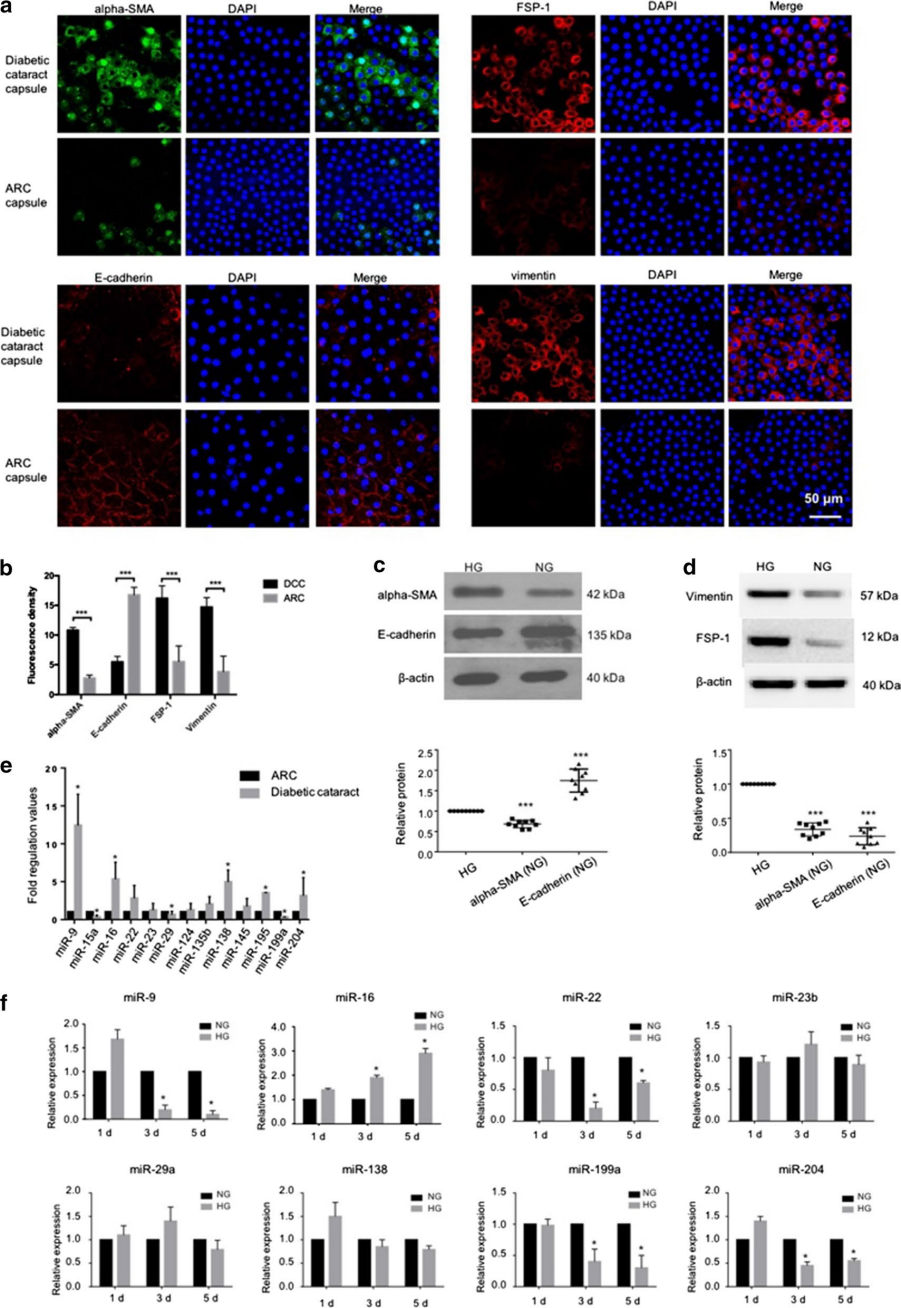
EMT and miRNA expression changes in human diabetic cataract and hyperglycemic LECs. **a**, **b** Immunofluorescence analysis was performed to detect the protein expression of EMT markers in capsules. The staining intensity of alpha-SMA, FSP-1 and vimentin expression increased in capsule of diabetic cataract than that of ARC, and E-cadherin expression decreased (n = 6). **c**, **d** The protein expression of EMT markers detected by western blot. An increased expression of alpha-SMA, FSP-1 and vimentin, while decreased expression of E-cadherin in HG conditions could be found (n = 9). **e** The expression of miRNAs was detected by RT-qPCR. MiR-9, miR-16, miR-138, miR-195, miR-204 were upregulated significantly, while miR-15a, miR-29 and miR-199a were downregulated in the diabetic cataract capsules (n = 9). **f** In the HG-treated LECs, miR-9 was upregulated firstly and then downregulated, while the expression of miR-16 and miR-29 were increased with HG exposure time. The expression of miR-22 and miR-199a were downregulated after 3 days of HG treatment (n = 3) (*P < 0.05; ***P < 0.005). *EMT* epithelial-to-mesenchymal transition, *LECs* lens epithelial cells, *DCC* diabetic cataract capsule, *ARC* age related cataract, *HG* high glucose

To illustrate the role of EMT in diabetic cataract formation, the features of alpha-smooth muscle actin (alpha-SMA), E-cadherin, FSP-1 and vimentin were detected. The capsules were collected from patients with diabetes mellitus or age-related cataract. As far as we can collect, the Table 1 exhibited the history (length) of diabetes, mean glucose and HbA1c of the patients enrolled in this study. As shown in Fig. 1a, b, the expression of alpha-SMA in diabetic cataract capsule (DCC) cells was higher than that in ARC cells, while the level of E-cadherin was higher in ARC capsules. Furthermore, the expression of FSP-1 and vimentin was found enhanced in diabetic cataract capsule cells compared to that in ARC cells. At the same time, EMT level of LECs in HG group in vitro was detected by western blot. The results revealed that expression level of alpha-SMA in HG-treated cells was significantly higher, while E-cadherin was inhibited compared with that in NG group (Fig. 1c). Moreover, the levels of vimentin and FSP-1 were increased in LECs exposed to high glucose (Fig. 1d). These results indicated that EMT was activated and participated in the diabetic cataract formation in either HG-conditioned LECs or diabetic patients.

**Table 1:** The history (length) of diabetes, mean glucose and HbA1c of the patients

	NC	DC
History of diabetes	–	11.28y (3y–32y)
Mean blood glucose level (mmo/L)	4.46 (3.56–5.07)	7.96 (4.83–11.36^a^)
HbA1c (%)	–	7.12 (6.8–10.1)

*NC* normal cataract, *DC* diabetic cataract

^a^For patients with the glucose over 8.3 mmo/L, alimentary control, sports and drugs regulation were taken to modulate the glucose less than 8.3 mmol/L for further cataract operations

To investigate the changes of miRNAs expression in diabetic cataract, real-time qPCR was performed on cataract capsules and LECs cultured under HG exposure for different periods. The results obtained from 30 capsules from each group showed that miR-9, miR-16, miR-138 and miR-195 were upregulated significantly (about 5 times), while miR-15a, miR-29 and miR-199 were downregulated in the diabetic cataract capsules (Fig. 1e). In HG-treated LECs, miR-9 was upregulated on the first day and then downregulated on the following days, while the expression of miR-16 and miR-29 increased with HG exposure time prolonging. The expression of miR-22 and miR-199a were downregulated after 3 days of HG treatment (Fig. 1f). These changes suggested that these miRNAs might be involved in the pathogenesis and progression of diabetic cataract.

### MiR-199a directly targeted SP1

**Fig. 2:**
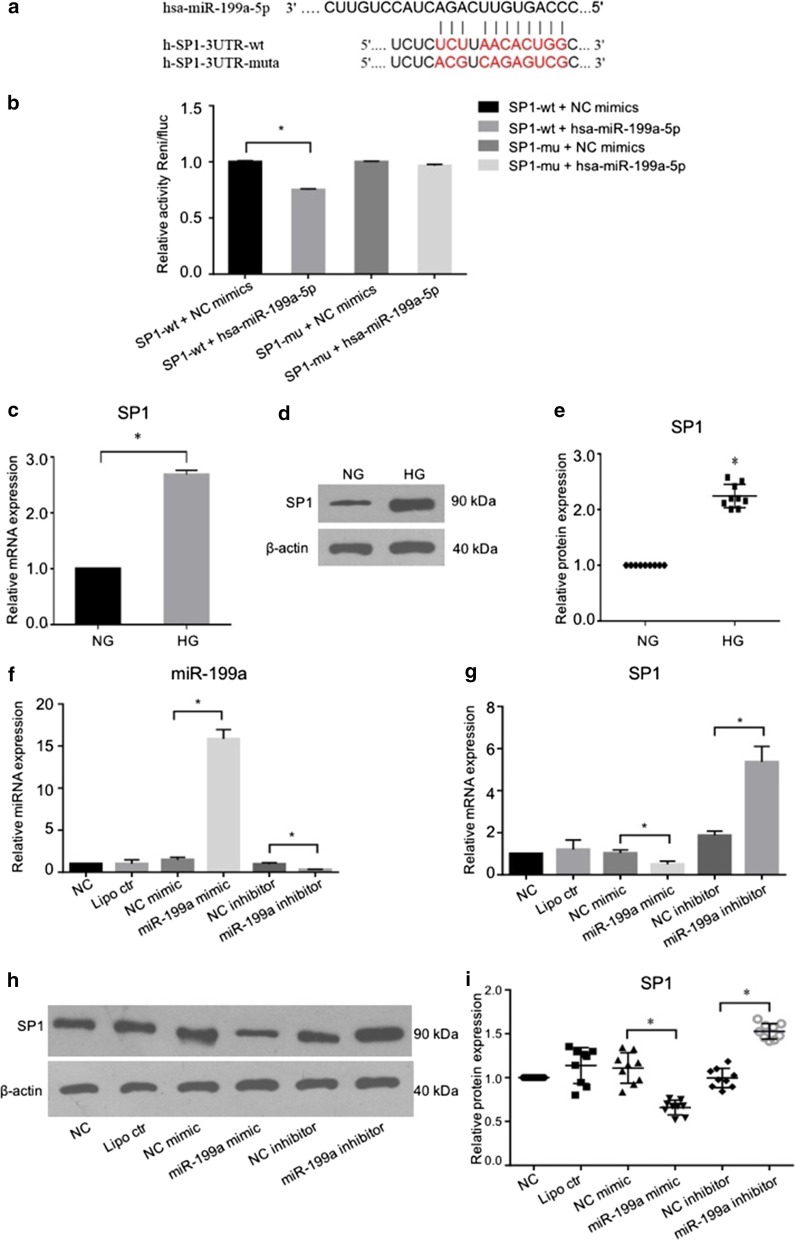
SP1 was the direct target of miR-199a. **a** Bioinformatics-based target analysis showed that SP1 is a potential target of miR-199a. **b** Luciferase reporter assay showed that the luciferase activity of SP1 3′UTR-wt significantly decreased with miR-199a mimic transfection, comparing to that of the NC mimic or SP1 3′UTR-mutant group (n = 3). **c–e** In the HG-treated cells, the mRNA and protein expression of SP1 increased significantly compared to NG control cells (n = 9). **f** The level of miR-199a was increased with miR-199a mimic transfection and decreased in the inhibitor group, compared with the normal cells, or negative control mimic/inhibitor. Meanwhile, lipofectamine (Lipo Ctr) did not affect miR-199a expression (n = 3). **g–i** SP1 mRNA and protein levels were downregulated significantly with the miR-199a mimic transfection, while they were upregulated when miR-199a was inhibited (n = 9). *SP1* specific protein 1, *HG* high glucose, *Lipo Ctr* only lipofectamine treated as the lipofectamine control. *P < 0.05

It was found that miR-199a was decreased in diabetic cataract and hyperglycemic LECs, thus the regulatory role of miR-199a was further explored. By analyzing the miRNA target with microRNA.org, and TargetScan, SP1 was predicted to be a potential target of miR-199a-5p (Fig. 2a). Dual luciferase reporter assay was performed to identify whether the 3′-UTR of SP1 mRNA was a binding target of miR-199a-5p. When the SP1 3′UTR-wt and miR-199a mimic were transfected together, the luciferase activity was significantly inhibited, compared to that of the NC mimic or SP1 3′UTR-mutant group, suggesting that miR-199a-5p could directly target the SP1 (Fig. 2b).

Furthermore, the regulatory effect of miR-199a-5p on SP1 was detected in vitro in HG-exposed LECs. In HG-treated cells, the SP1 mRNA and protein expression increased significantly, compared to NG-treated cells (Fig. 2c–e). After transfection, miR-199a expression enhanced significantly in the miR-199a mimic transfected group and decreased in the inhibitor group, comparing with the normal cells, and cells transfected with negative control mimic or inhibitor, while Lipofectamine did not affect miR-199a expression (Fig. 2f). SP1 mRNA and protein levels were inhibited significantly after the miR-199a mimic transfection, while downregulation of miR-199a by inhibitor enhanced the SP1 expression (Fig. 2g–i). These results confirmed that SP1 was directly bound and regulated by miR-199a-5p. Consecutively, this modulation might influence the downstream targets of SP1 to affect the diabetic cataract progression.

### MiR-199a repressed EMT in hyperglycemic LECs

**Fig. 3:**
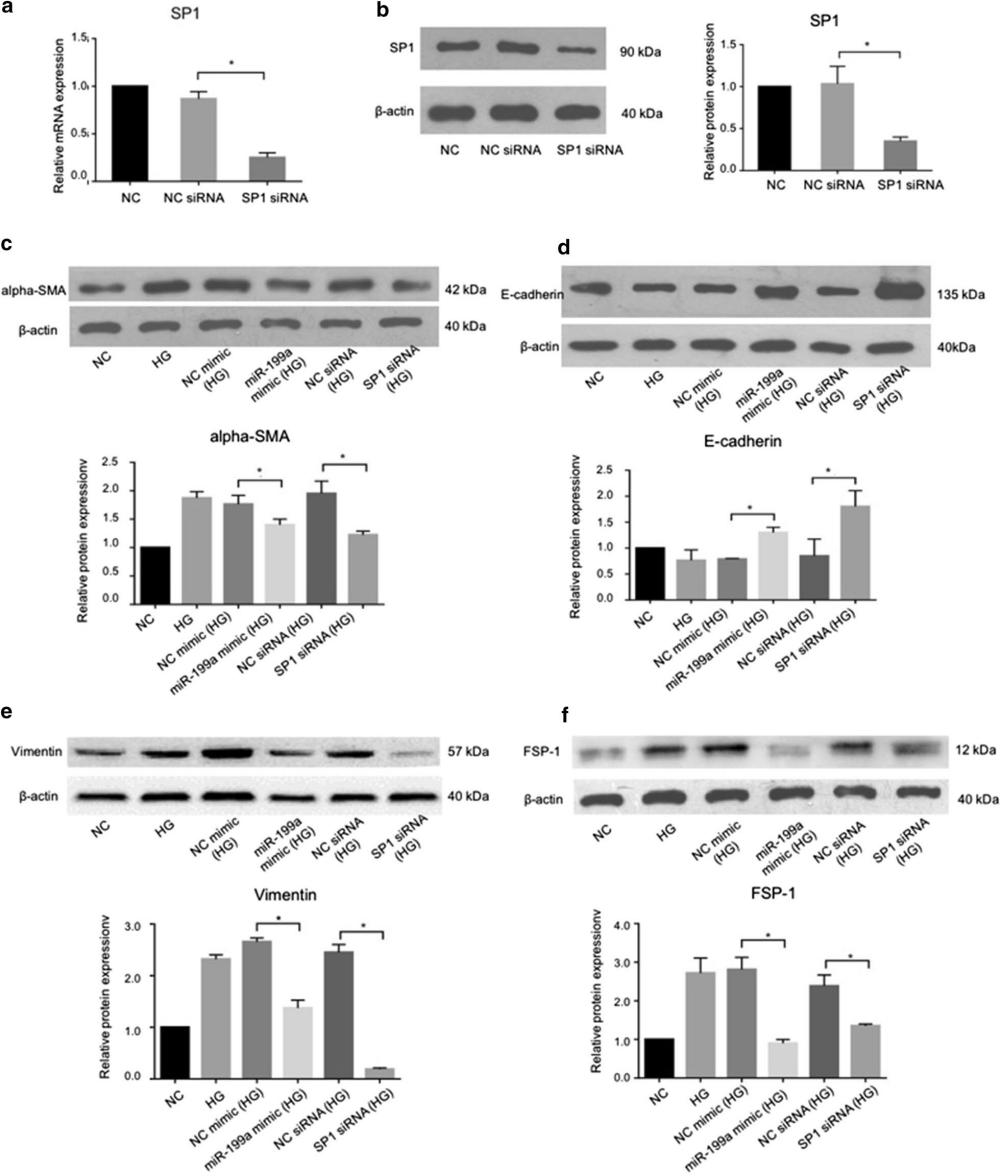
MiR-199a repressed EMT through SP1 in hyperglycemic LECs. **a**, **b** SP1 mRNA and protein expression were significantly decreased by SP1 siRNA transfection (n = 6). **c–f** After miR-199a mimic transfection, the protein expression of alpha-SMA, vimentin and FSP-1 was downregulated compared with NC mimic in HG condition, while the E-cadherin was upregulated. When SP1 siRNA was transfected, alpha-SMA, vimentin and FSP-1expression decreased while E-cadherin expression increased (n = 6). *SP1* specific protein, *HG* high glucose, *LECs* lens epithelial cells. *P < 0.05

To analyze the potential effect of miR-199a on EMT in diabetic model in LECs, the cells transfected with miR-199a mimic were cultureed in HG condition for 5 days, prior to the protein levels of E-cadherin and alpha-SMA were detected. As illustrated in Fig. 3, after miR-199a mimic transfection, the expression of alpha-SMA protein was downregulated, compared with NC mimic under HG conditions, while the E-cadherin was upregulated. These revealed that the upregulation of miR-199a might repress EMT in the diabetic cataract model in vitro. Moreover, the expression level of alpha-SMA was decreased while the expression of E-cadherin was increased with the SP1 silencing in HG-treated cells. Accordingly, it indicated that miR-199a could directly target SP1 to inhibit EMT in diabetic LECs.

## Discussion

In the present study, we identified potential miRNAs participating in EMT during diabetic cataract progression. In diabetic cataract capsule and hyperglycemic LECs, miR-199a-5p was downregulated. miR-199a repressed EMT in a SP1-dependent way in HG-conditioned LECs in vitro.

Diabetic cataract is a common chronic eye complication associated with DM, the incidence of which is arising. Increasing miRNAs aberrantly expressed during the cataract development have been identified, while the miRNAs in diabetic cataract formation are catching more attention in the mechanism studies (Varma et al. 2012; Zhang et al. 2017a, 2017b; Zeng et al. 2017). To identify the differentially expressed miRNAs in diabetic cataract, miRNA expression was analyzed in the capsules of DM patients and in cells exposed to hyperglycemia in vitro. Among the identified miRNAs, miR-9, miR-16, miR-138 and miR-195 were upregulated, while miR-15a, miR-29 and miR-199 were downregulated in the diabetic cataract capsules. In HG-treated LECs, miR-199 showed a same descending trend and miR-16 was increased consistently as that in the capsules, while miR-9, miR-22 and miR-204 exhibited opposite expression changes, implying that the same miRNAs might have different expression levels and play opposite roles in different phases of diabetic cataract progression. The underlying reason for these findings could be that the pathologic changes associated with diabetes progression exert distinct effects on miRNA biogenesis, resulting in either upregulation or downregulation at different stages of diabetic cataract. Therefore, the application of miRNAs in clinical therapy should be based on the modulation of miRNAs for which stable and confirmed changes in expression have been reported.

Among the miRNAs studied, miR-199a was downregulated during diabetic cataract development both in vitro and in vivo. miR-199a was previously reported to be related to cell migration, metastasis and angiogenesis in cancer cells, as well as the modulation of inflammatory microenvironment, and it can be downregulated by hypoxic stimuli (Joshi et al. 2014; Chen et al. 2008). Additionally, miR-199a was shown to target PARP-1 and activate the ERK1/2 pathway to promote IL-10 production, suggesting that miR-199a may be a potential therapeutic target of systemic lupus erythematosus (Su et al. 2019). In models of DM, miR-199a was also found to be increased along with the diabetic retinopathy development and paly a cross-talk role between VEGF and hypoxia-inducible factor 1α (Gong et al. 2017; Ling et al. 2013). In addition, miR-199a was suggested to be a potential biomarker for diabetic nephropathy which targets Zinc Finger E-box-Binding Protein 1 (Meng et al. 2018). Moreover, functional study revealed that miR-199 can regulate the proliferation and EMT of the triple negative breast cancer together with miR-214 (Cantini et al. 2019). Therefore, the role of miR-199a in the regulation of EMT in the diabetic cataract model aroused investigation in this study.

**Fig. 4:**
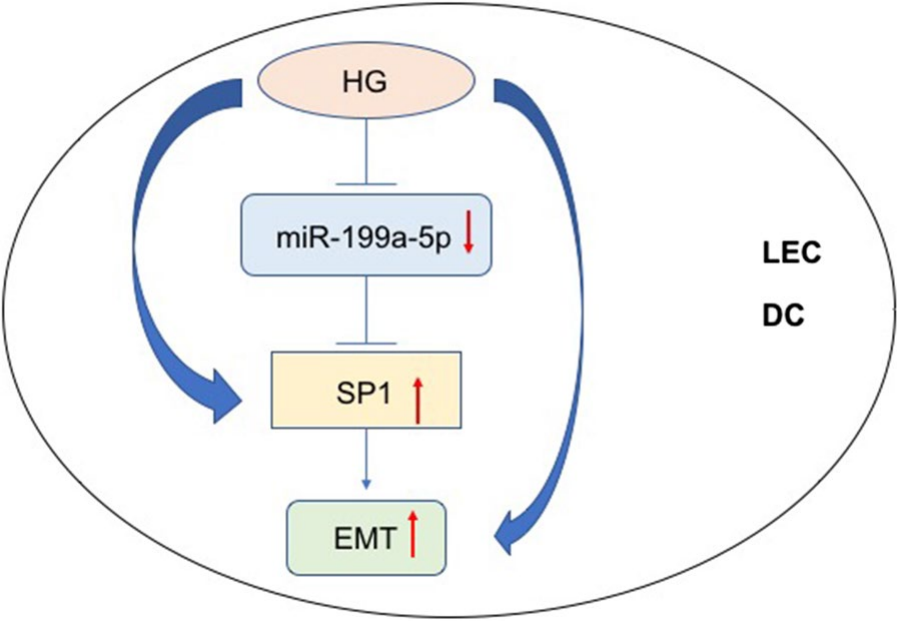
MiR-199a participated in the suppression of EMT by targeting SP1 gene in diabetic cataract

Different mechanisms have been proposed for the pathogenesis of diabetic cataract, including polyol pathway related aldose reductase mechanism, osmotic stress, oxidative stress, and autoimmune theories, and all these are involved in LECs apoptosis and hydropic lens fibers (Kiziltoprak et al. 2019; Kador et al. 2016; Mulhern et al. 2007; Papadimitriou et al. 2016). EMT, which participates in cataract formation by affecting LECs differentiation and in posterior capsular opacification (PCO) formation by affecting cell motility and migration, play a role in diabetes-related fibrosis and diabetic cataract development (Zhang et al. 2017a; Taiyab et al. 2016; Wei et al. 2015). Via the prediction of microRNA.org, and TargetScan, SP1 is highly conserved to be the target of miR-199a. SP1 is an important transcription factor for housekeeping genes. Overexpression of SP1 is related to abnormal differentiation of cells, while SP1 downregulation aggravates endoplasmic reticulum (ER) stress and unfolded protein response (Safe and Abdelrahim 2005; Dauer et al. 2017). SP1 could modulate EMT, the proliferation and invasion of cancer cells by regulating related genes (Kim et al. 2019; Sun et al. 2018). Therefore, miR-199a may modulate SP1 to affect EMT, and then regulate the progression of diabetic cataract. In our study, EMT was confirmed involved in diabetic cataract capsules and HG-conditioned LECs where overproduction of alpha-SMA and downregulation of E-cadherin was found. Luciferase assay confirmed that SP1 was regulated by miR-199a directly in LECs. The silencing of SP1 repressed alpha-SMA transcription and increased E-cadherin transcription to inhibit EMT in hyperglycemic LECs. These revealed that the overexpression of miR-199a, as well as SP1 downregulation, could inhibit EMT in HG- conditioned LECs. Overall, the network of miR-199a-SP1-EMT played a role in the progression of diabetic cataract (Fig. 4).

## Conclusions

In conclusion, we demonstrated that miR-199a was downregulated in LECs of diabetic cataract model in vitro and lens capsules in vivo. MiR-199a participated in the suppression of EMT by targeting SP1 gene in LECs. These results provide new insights into the molecular mechanisms underlying the diabetic cataract development, and meanwhile, miR-199a may be a new therapeutic target for diabetic cataract.

## Fundings

This work was supported by the Special Fund for Health of Jilin Province [No. 201817421534], the National Natural Science Foundation of China [No. 82000920] and the Natural Science Foundation of Jilin Province [No. 20180520118JH].

## Supplementary Information

**Additional file 1: Figure S.A **SRA cells-monolayer under normal conditions.

**Additional file 2: Figure S.B **SRA cells-characteristic of mesenchymal cells exposed to high glucose for 5 days.

## Availability of supporting data

The data used to support the findings of this study are available from the corresponding author upon request.
