# Supplementary figures and images for: microRNA-199a-5p regulates epithelial-to-mesenchymal transition in diabetic cataract by targeting SP1 gene
Source: Mol Med. 2020 Dec 4;26:122. doi: 10.1186/s10020-020-00250-7 (PMC7718685; doi:10.1186/s10020-020-00250-7)

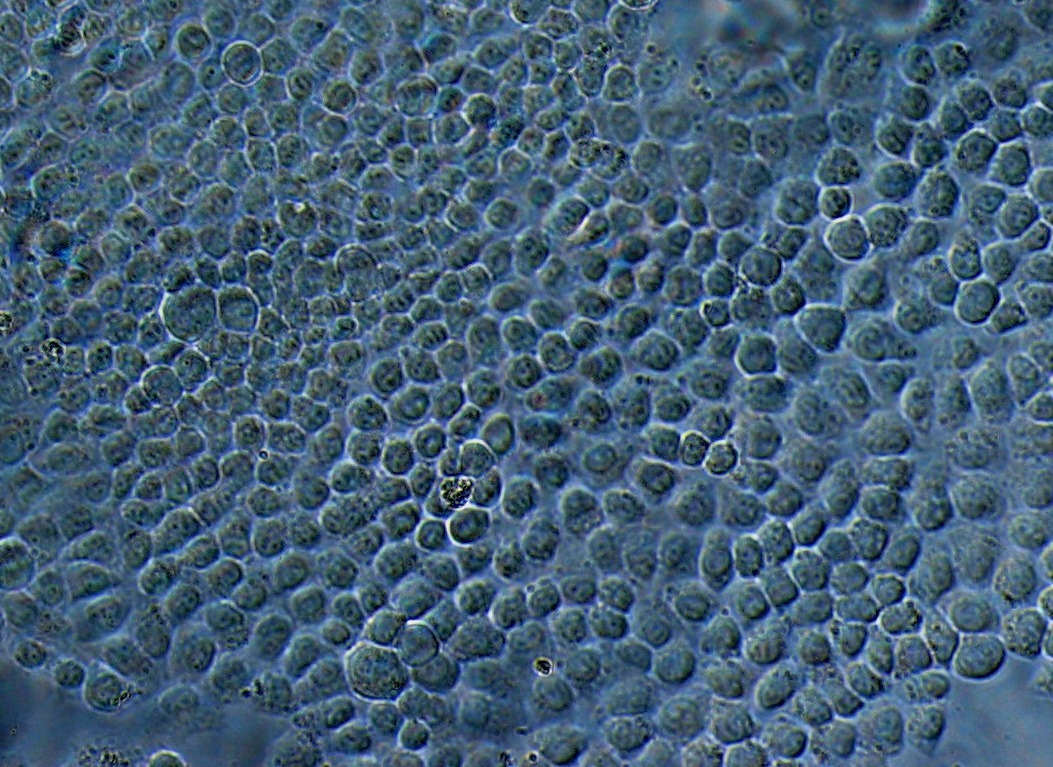

Supplement: Supplementary file 1 — Additional file 1: Figure S.A SRA cells-monolayer under normal conditions. [file 10020_2020_250_MOESM1_ESM.png]

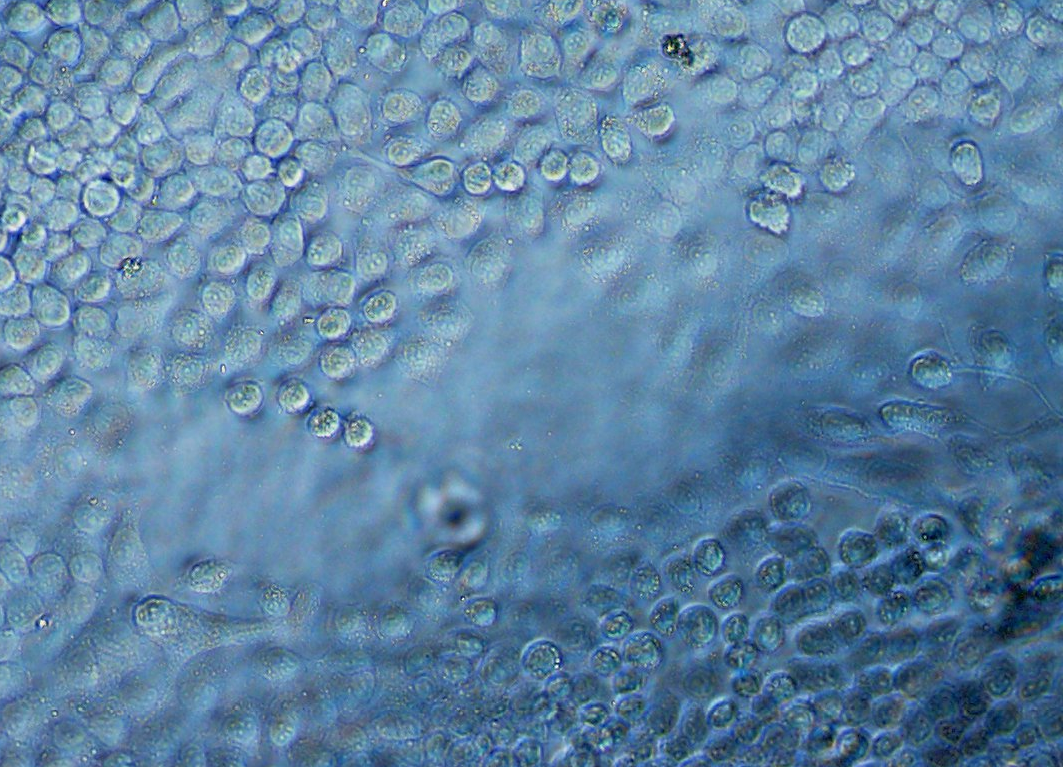

Supplement: Supplementary file 2 — Additional file 2: Figure S.B SRA cells-characteristic of mesenchymal cells exposed to high glucose for 5 days. [file 10020_2020_250_MOESM2_ESM.png]
